# Association between high arterial oxygen tension and long-term survival after intracerebral hemorrhage

**DOI:** 10.1186/cc14545

**Published:** 2015-03-16

**Authors:** M Fallenius, R Raj, M Reinikainen, S Bendel, MB Skrifvars

**Affiliations:** 1 Helsinki University Hospital, Helsinki, Finland; 2 North Karelia Central Hospital, Joensuu, Finland; 3 Kuopio University Hospital, Kuopio, Finland

## Introduction

Liberal use of oxygen after brain insults remains controversial [1,2]. We studied whether high arterial oxygen tension (PaO_2_) is associated with decreased long-term survival in patients with spontaneous intracerebral hemorrhage (ICH) treated in the ICU.

## Methods

Data on primary admissions for adult patients (>18 years) treated for ICH in Finnish ICUs between 2003 and 2012 were collected from a nationwide ICU database. Patients were divided into three groups according to the PaO_2_ value associated with the lowest measured PaO_2_/ FIO_2_ ratio during the first 24 hours after ICU admission. High arterial oxygen tension was defined as PaO_2_ >19.9 kPa; intermediate as PaO_2 _13 to 19.9 kPa; and low as PaO_2_ <13 kPa. The primary outcome was 6-month mortality.

## Results

Of the 3,033 patients, 63% (*n *= 1,923) had low PaO_2_, 29% (*n *= 892) intermediate PaO_2_, and 7% (*n *= 218) high PaO_2_. Forty-nine percent of the patients died during the 6-month follow-up. Of these, 75% died before discharge from hospital. Univariate analysis showed that 6-month mortality was higher in the high PaO_2_ group (61%) compared with the intermediate and low PaO_2_ groups (52% and 46% respectively, *P *< 0.001). Multivariate analysis, however, showed no statistically significant correlation between high PaO_2_ and mortality (with the low PaO_2_ group as the reference category, odds ratio for death (OR) for high PaO_2 _= 1.10, 95% confidence interval (CI) = 0.76 to 1.58 and for intermediate PaO_2_ = 0.96, 95% CI = 0.78 to 1.17).

## Conclusion

High PaO_2_ is not predictive of 6-month mortality in patients treated for spontaneous ICH in the ICU. Therefore, targeting higher PaO_2_ values appears to be a safe approach in order to avoid hypoxemia.
